# Dietary Isoflavone Aglycons from Soy Germ Pasta Improves Reproductive Performance of Aging Hens and Lowers Cholesterol Levels of Egg Yolk

**DOI:** 10.3390/metabo12111112

**Published:** 2022-11-15

**Authors:** Kenneth D. R. Setchell, Evangelia Mourvaki, Carlo Clerici, Simona Mattioli, Gabriele Brecchia, Cesare Castellini

**Affiliations:** 1 Department of Pathology and Laboratory Medicine, Cincinnati Children’s Hospital Medical Center, Cincinnati, OH 45229, USA; 2 Department of Pediatrics, University of Cincinnati College of Medicine, Cincinnati, OH 45229, USA; 3 Department of Clinical and Experimental Medicine, Gastroenterology and Hepatology Section, University of Perugia, S. Andrea delle Fratte, 06156 Perugia, Italy; 4 Department of Agricultural, Environmental and Food Science, University of Perugia, Borgo XX Giugno 74, 06124 Perugia, Italy; 5 Department of Veterinary Medicine, University of Milano, Via dell’Università, 6, 26900 Lodi, Italy

**Keywords:** isoflavones, equol, soy germ, poultry, eggs, cholesterol

## Abstract

This study compared dietary isoflavone aglycones with the glycoside conjugates in a novel model of postmenopausal status, the aging domestic hen (*Gallus gallus domesticus*), to determine the effects on reproductive performance, cholesterol levels, and nutritional quality of eggs laid. Hens, 18 mo old, were randomized into four groups (*n* = 10/group) and fed for 28 d a conventional poultry corn/soymeal diet (Control), or diets supplemented with isoflavone glycosides from soy germ (diet A), isoflavone aglycons from a soy germ pasta (diet B), or conventional pasta lacking isoflavones (diet C). The egg-laying rate was recorded daily, plasma isoflavones and cholesterol were measured, and the nutritional composition of the eggs was determined. Egg-laying declined over a 4-week period in hens in the Control group and those fed isoflavone glycosides (diets A and C), whereas hens fed isoflavone aglycons (diet B) significantly increased their egg-laying efficiency. The total egg count and egg yield were significantly higher in hens fed isoflavone aglycons, and their plasma cholesterol concentrations were lower and the eggs laid had a 30% lower yolk cholesterol content. None of these effects were observed with diets containing similar levels of isoflavone glycosides. These studies recapitulate the clinical effects of soy germ pasta enriched with isoflavone aglycons and lend support to the greater efficacy of a diet rich in isoflavone aglycons.

## 1. Introduction

The discovery of *S*-(-)equol in human urine happened more than 40 years ago [1], and its association with soy-based diets [2] led to the hypothesis that soy foods and specifically nonsteroidal soy isoflavones play a protective role against many hormone-dependent diseases [3]. *S*-(-)equol was shown to be produced by intestinal microflora [3,4], of which many strains and species have been shown to metabolize its precursor, daidzin/daidzein [5,6,7,8,9,10,11]. Not all adults are able to produce *S*-(-)equol when consuming soy-based foods [3,12,13,14,15,16,17,18,19,20], an observation that led to the proposal that the conversion to *S*-(-)equol, with its greater biological activity, may be an important factor in determining the efficacy of a soy food diet—the so-called “equol-hypothesis” [21]. A number of dietary factors have been associated with the ability to produce *S*-(-)equol [13,22,23,24,25,26,27], but a consensus on which are the most important remains elusive. What is clear is that the ability of an individual to produce *S*-(-)equol requires the presence of specific equol-producing bacteria and optimal conditions in the gastrointestinal tract for the hydrolysis, reduction and deoxygenation reactions to take place to convert daidzin to *S*-(-)equol [5]. The first reaction, the hydrolysis of the glucose moiety of daidzin, which is one of the two principal isoflavone forms in most soy foods, is crucial because isoflavone glycosides, unlike aglycons, do not cross the enterocyte and are, therefore, not bioavailable [28]. Furthermore, these conjugated isoflavones are not biologically active. We proposed that the higher proportion of “equol-producers” consistently reported for adults living in Asia may be because of the intake of greater proportions of the biologically active aglycons in the soy foods consumed [5,29]. This contention was supported by our studies of a novel soy germ pasta that coincidentally contained a high proportion of isoflavone aglycons (74%) because of a unique reaction between β-glucosidase in semolina (durum wheat) and the isoflavone glycoside, daidzin, which is the major isoflavone of the soy germ that occurred during manufacture [30]. Dietary intervention studies of this novel soy germ pasta showed significant effects of reducing lipids and improving a number of cardiovascular risk factors in patients with hypercholesterolemia [30] and type 2 diabetes [31], while, interestingly, normalizing gastroparesis in diabetes patients with associated gastroparesis [32]. In these studies, 55–69% of the adults produced equol after consuming soy germ pasta [30], which is similar to the 50–70% reported frequency for Asians consuming soy foods [15,16,17,25].

The objective of this study was to further illustrate the efficacy of isoflavone aglycons over the glycoside conjugates using a novel model of postmenopausal status, the aging domestic hen (*Gallus gallus domesticus*), a species that exclusively produces *S*-(-)equol [33,34,35]. We studied this species in a period of natural decline in egg-laying efficiency [36], a period that coincides with a decline in estrogen production. Consistent with the lipid-lowering effect of soy germ pasta in hypercholesterolemic patients [30], we hypothesized that isoflavone aglycons, delivered in this food, would reduce the circulating cholesterol levels in hens in an inverse relationship to *S*-(-)equol concentrations, and in doing so, would result in the production of eggs with an improved quality through a lower yolk cholesterol content. Furthermore, we hypothesized that such effects would not be observed by comparable levels of conjugated isoflavones in the diet, and if proven, would further support the advantages to human health of soy foods rich in isoflavone aglycons, commonly consumed in Asian countries [37,38,39].

## 2. Materials and Methods

### 2.1. Diets

All diets were formulated according to the recommended National Research Council nutrient requirements of Leghorn hens [40] and were based on the published literature [41]. Four different diets were used, as indicated in Table 1. The diet normally fed to hens is a commercial poultry corn/soymeal diet (Mignini s.p.a, Petrignano, PG, Italy) made from mainly extruded soymeal blended with corn meal (Table 1). The Control diet consequently contained isoflavone glycosides derived from the soy protein. Diet A consisted of the Control diet, to which 0.9% soy germ was added. The soy germ naturally contained 25 mg/g of total isoflavones, predominantly as isoflavone glycosides. Diet B consisted of the Control diet formulated to contain predominantly isoflavone aglycons. This was possible by substituting the wheat bran and corn meal with a finely ground dried soy germ pasta (Aliveris s.r.l., Perugia, Italy). The nutritional composition of the soy germ pasta, which has been reported in detail previously [30], comprised 38.5–41% protein, 22–28% carbohydrates, 14–18% fat, 4% moisture, 2–4% fiber, 3–5% saponins, 0.3–0.6% lecithin and 0.015–0.025% tocopherol, and naturally contained about 41.25 mg/100 g of total isoflavones, of which 74% were in the aglycon form as a result of hydrolysis of the isoflavone glycosides during the manufacture of the pasta [30]. The soy germ pasta was milled using a Cyclotec 1093 sample Tecator to a particle size of about 1 mm to be consistent with the other ingredients of the poultry diet. Diet C was the Control diet to which the same proportion of similarly finely ground conventional wheat (durum wheat semolina) pasta, lacking any soy germ or isoflavones, was added. The composition of the soy germ pasta used as the dietary source of isoflavone aglycons has been described in detail elsewhere [30]. The amount of soy germ contained in diet B from the added ground soy germ pasta was equivalent to 1.06%, which was slightly higher than for diet A (0.9%), but this small difference was not considered to be physiologically relevant. Independent of the chemical form of the isoflavones, the amount of total isoflavones added to the corn/soymeal soybean diets was 225 mg/kg, measured as aglycon equivalents, thus approximately doubling the total isoflavone content of the typical commercial poultry Control diet. All four diets were isonitrogenous and isocaloric and of similar micro- and macrocomposition; details of the ingredient and nutrient contents of these diets are summarized in Table 1.

### 2.2. Study Design

In this 28-day study (28 d), White Leghorn hens (*Livornese Bianca*, *n* = 40) aged 18 months [42], from the same hatch and in the late stage of their laying cycle (only 37.8% of the hens were efficiently laying eggs), were randomly assigned to the 4 dietary groups (*n* = 10 birds/group). All diets and water were provided ad libitum for 28 consecutive days and feeding was carried out daily during the morning and monitored by weighing the feeders and evaluating the residue. The birds were individually housed in enriched cages (one hen per cage) and maintained under the same conditions of light/dark cycles, temperature and humidity. The study was conducted at a commercial farm. All procedures involving the care and handling of animals met the National Guidelines for Animal Care. The hens were weighed and body weights recorded at the beginning (baseline) and end (final) of the 28 d study period, and the average group body weights were calculated. Average body weight gain and total feed consumption were calculated for each group. The baseline laying performance of the hens was measured for 2 weeks prior to the randomization of the different diets, and thereafter, weekly. All eggs produced by the hens were collected, counted, registered, and weighed daily. The actual egg yield for each dietary group was determined by multiplying the number of eggs by the egg weight. The egg-laying rate was calculated by dividing the total number of laid eggs by the days and by the number of animals belonging to each dietary group. At the end of the study, the hens were sacrificed and approximately 2.5 mL of blood was obtained from the jugular vein of each hen. The blood was collected in EDTA-coated tubes, samples were centrifuged at 2500 rpm for 15 min, and the plasma was separated and stored frozen at −20 °C for analysis of isoflavone [43] and cholesterol concentrations.

### 2.3. Evaluation of Egg Quality and Characteristics

Ten eggs per diet group were randomly collected in the last week for the measurement of egg composition. The percentages of albumen, yolk and shell were calculated relative to the whole egg weight. The albumen/yolk ratio was also measured. Albumen consistency was tested by calculating the Haugh unit (HU) value of each egg, defined as follows: HU = 100 log [H − g^0.5^ (30W^0.37^ − 100)/100 + 1.9], where H is the maximum height of the thick albumen in millimeters, W is the weight of the egg in grams and g is a constant (32.2) related to the constant of gravitation. Yolk color intensity was established using the Roche color fan scale (1–15) [44]. The chemical composition of egg yolks (*n* = 10/group) was determined using the procedures of the Association of Official Analytical Chemists [45].

### 2.4. Analytical Determination of Isoflavones and Cholesterol

The isoflavone composition of the four diets was determined by reverse-phase HPLC as described in detail previously [46]. Plasma concentrations of daidzein, genistein, glycitein and *S*-(-)equol, the metabolite of daidzein, were determined by mass spectrometry after solid-phase extraction and hydrolysis of the conjugates [14,43]. The isoflavone composition of the egg yolk, including *S*-(-)equol, was determined via mass spectrometry after extraction of isoflavones from 5 g samples of egg yolk by refluxing in 80% aqueous ethanol (25 mL) for 30 min. After filtering the sample through a Whatman No. 1 filter paper, the ethanolic extract was made with up to 50 mL of absolute ethanol and 5 mL taken to dry under a stream of nitrogen. This extract was then further processed, as described for the plasma samples [14,43].

Total plasma cholesterol concentration and egg-yolk cholesterol content were determined by a routine colorimetric method (Hitachi U-2000, set at 500 nm and 405 nm, respectively) using a commercial enzymatic kit (Boehringer, Mannheim, Germany; Manual for Food Analysis, 1995).

### 2.5. Statistical Analysis

The effect of the diets on the hen’s egg production performance, isoflavone and cholesterol concentrations, and on measures of egg quality were analyzed by a one-way ANOVA, and significant differences were determined using the post hoc Bonferroni test at the level of *p* < 0.05. The Pearson regression was used to evaluate significant correlations between the variables (STATA, 2015, StataCorp, College Station, Texas TX77845).

## 3. Results

### 3.1. Isoflavone Content of Diets

An HPLC analysis of the commercial Control diet revealed a total isoflavone content of 33.6 mg/100 g diet (Table 2), which was consistent with the expected value based on published levels of isoflavones in soymeal [47,48,49]. Diet A, which was supplemented with 0.9% soy germ, and diet B with ground soy germ pasta both had approximately double the total isoflavone content of the Control diet (*p* < 0.05), the increase being accounted for by isoflavones derived from either soy germ (mainly glycosides [49]), or ground soy germ pasta (mainly aglycons [30]). Qualitatively, all the diets contained mainly glycoside or aglycon forms of genistein and daidzein and a low proportion of glycitein. The relative proportion of total daidzein, genistein and glycitein forms in diets A and B was similar, and expectedly different from that of the Control diet and diet C (Table 2). The isoflavone conjugation pattern of the diets also differed. The Control diet and diets A and C contained predominantly isoflavone glycosides (>95%), whereas diet B containing the ground soy germ pasta had a >10-fold higher proportion of isoflavone aglycons than the other three diets.

### 3.2. Influence of Dietary Isoflavones on Egg-Laying Performance of Hens

**Figure 1:**
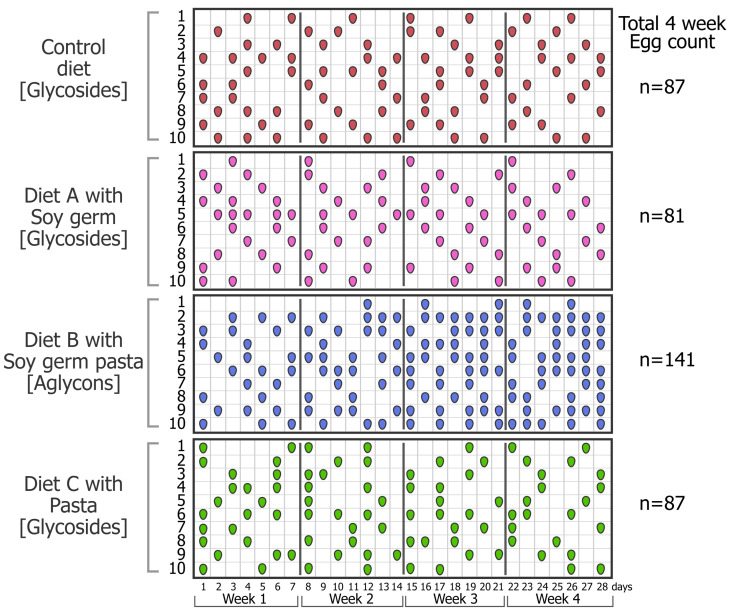
An “egg-array” depicting the daily laying performance of each hen (*n* = 10/group) monitored over the 28-d period for aging White Leghorn hens fed a commercial corn/soymeal diet (Control), the same diet supplemented with soy germ to increase the isoflavone glycoside content (diet A), the same diet with added milled soy germ pasta containing predominantly isoflavone aglycons (diet B) and the same diet with added milled commercial pasta lacking isoflavones (diet C). The grid depicts each day an egg was laid by the individual hens within the diet group.

**Figure 2:**
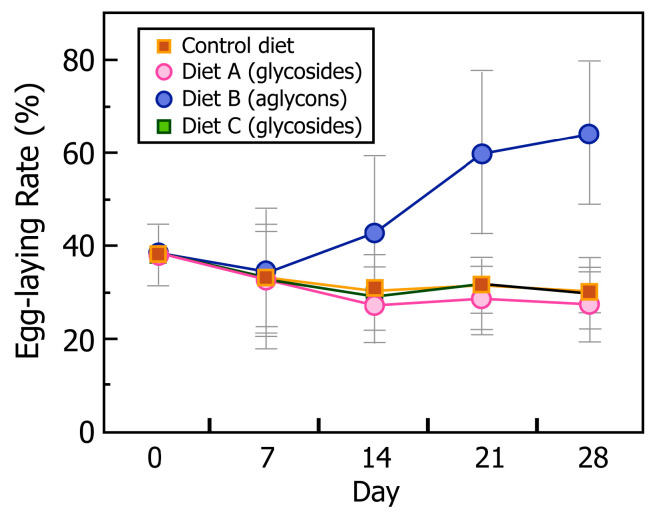
Graph showing the mean ± SD egg-laying efficiency of aging White Leghorn hens measured over a 28-d period for groups of hens fed a commercial corn/soymeal diet (Control), the same diet supplemented with soy germ to increase the isoflavone glycoside content (diet A), the same diet with added milled soy germ pasta containing predominantly isoflavone aglycons (diet B) and the same diet with added milled commercial pasta lacking isoflavones (diet C).

Baseline egg laying (defined as the proportion of hens that were laying eggs), assessed over a 2-week period prior to randomization of the four dietary groups, was 37.8 % (*n* = 40), and the average number of eggs laid per group during this period was approximately four eggs/hen. After randomization into the four diet groups (*n* = 10/group), the egg-laying performance was similar among groups at baseline. Figure 1 shows a grid displaying the daily egg-laying performance of each individual hen, documenting each day an egg was laid—we refer to this as an “egg-array”. The egg-laying performance of the hens declined over the 4-week period of study in hens fed the Control diet and diets A and C, which all had mainly isoflavone glycosides, and the decline was not significantly different from the baseline among the groups over this 4-week period. By contrast, as is evident in this egg-array, the laying performance of hens fed diet B containing isoflavone aglycons from ground soy germ pasta progressively increased each week after the first week, so that in the final week, 64% of the hens were laying eggs (Figure 2). This difference was statistically significant when compared with the baseline and also when compared with other groups at 4 weeks.

The total number of eggs produced by hens fed the Control diet and diets A and C was similar over the 4-week study (Figure 1). Although the hens fed diet A produced eggs that, on average, were slightly heavier, the overall total egg yield (expressed as total number of eggs produced multiplied by the average egg weight) from hens in these three groups was similar (Table 3). By contrast, hens fed diet B with isoflavone aglycons produced a significantly greater number of eggs (141 eggs) over the same period when compared to hens fed either the Control diet or diets A and C (87, 81 and 87 eggs, respectively). Although the average weight of the eggs produced was slightly lower, the overall total egg yield from hens fed diet B was significantly greater than hens fed the other diets.

Total feed intake over the 4-week study was notably higher in hens fed diet B (33.6 kg) compared with hens fed the Control diet (29.8 kg) or diets A and C (28.9 and 30.7 kg, respectively). Despite a much greater food intake by hens fed diet B, there were no significant differences in the final body weight of hens among the four groups (Table 3).

### 3.3. Effects of Dietary Isoflavones on Nutritional Composition of Eggs

The composition and nutritional content of the eggs, determined from the analysis of 10 randomly selected eggs from each diet group laid in the final week of the study, are summarized in Table 4. Major differences were found in the composition and quality of eggs produced by hens fed diet B, which was supplemented with isoflavone aglycons. Eggs produced by this group of hens had the highest % albumen content and albumen/yolk ratio compared with the other groups, and these differences were statistically significant. The edible mass and % shell weight of the eggs was similar among all groups, as was the quality of the eggs, measured in Haugh units (>76 HU). Significant differences were found in the egg-yolk color as measured on the Roche scale, with eggs from groups B and C being noticeably less yellow in color than eggs of the other two groups; however, analyses of the nutritional composition of the yolks showed no significant differences among the groups.

The egg cholesterol content was affected by the dietary treatment (Table 4). Strikingly, hens fed only the diet containing isoflavone aglycons (diet B) from soy germ pasta laid eggs with a significantly lower total cholesterol content when compared with eggs laid by hens in the Control group and those fed diets A and C (220 vs. 262, 266 and 275 mg/egg, respectively; *p* < 0.05), partly due to the lower proportion of yolk. Egg cholesterol content was positively correlated with the hen’s circulating plasma cholesterol concentration (y = 0.21x + 8.85; R^2^ = 0.77).

Measurement of the isoflavone content of the eggs revealed *S*-(-)equol as the major isoflavone. Traces of daidzein, genistein and glycitein were detected, but at levels too low to be accurately quantified. The average concentration of *S*-(-)equol was highest in the eggs laid by hens fed the aglycon-rich diet B (2.46 μg/egg). Eggs produced by hens fed the Control diet, which contained isoflavones from soybean meal, had low but measurable levels of *S*-(-)equol (0.69 μg/egg). Despite a similar intake of total isoflavones from diets A and B, the *S*-(-)equol content of the eggs from hens fed mainly isoflavone aglycons (diet B) was significantly higher than in eggs produced from hens fed the diets containing mainly isoflavone glycosides (diet A, 1.86 μg/egg). Diet C, containing conventional pasta and lacking isoflavones, yielded eggs with a similar *S*-(-)equol content (0.67 μg/egg) to that of the Control diet.

### 3.4. Effects of Dietary Isoflavones on Plasma Equol and Cholesterol Levels

**Figure 3:**
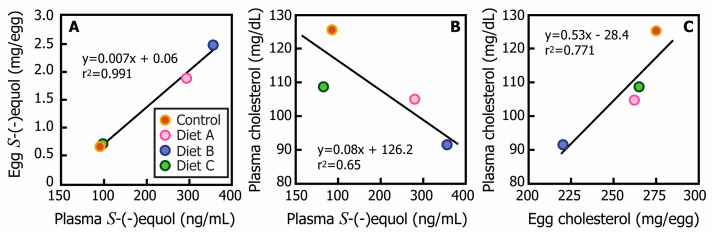
Plots showing (**A**) a positive correlation between the average egg plasma *S*-(-)equol content (μg/egg, *n* = 10) and average plasma *S*-(-)equol concentration (ng/mL, *n* = 10), (**B**) an inverse relationship between plasma cholesterol and plasma *S*-(-)equol concentration (ng/mL, *n* = 10), and (**C**) a positive correlation between plasma cholesterol concentration and egg-yolk cholesterol content of hens fed commercial corn/soymeal diet (Control), and the same diet with added isoflavone glycosides from soy germ (diet A), isoflavone aglycons from a soy germ pasta (diet B) or with added commercial pasta lacking isoflavones (diet C).

The mean plasma *S*-(-)equol concentration of hens fed diets A (294 ng/mL) and B (357 ng/mL) was significantly higher than hens fed the Control diet (98 ng/mL) or diet C (91 ng/mL). This difference was consistent with the higher total isoflavone content of the diets A and B. However, despite similar total isoflavone intakes of diets A and B, the plasma *S*-(-)equol concentration was much higher in hens fed the diet containing mainly isoflavone aglycons. There was a positive correlation between the mean plasma *S*-(-)equol concentration and the egg content of *S*-(-)equol (Figure 3). The mean plasma cholesterol concentration of hens fed this diet (91 ± 6 mg/dL) was significantly lower than for hens fed diet A (105 ± 6 mg/dL), diet C (108 ± 3 mg/dL) or the Control diet (127 ± 11 mg/dL), and an inverse relationship was observed between plasma cholesterol and plasma equol concentrations (Figure 3). The trend to lower plasma cholesterol concentrations in the hens was paralleled by the lower cholesterol content of the egg.

## 4. Discussion

The primary goal of this study was to determine whether the hypocholesterolemic and beneficial effects observed in clinical studies of a unique soy germ pasta [30,31] could be replicated in a novel animal model, the White Leghorn hen, and, moreover, to determine whether the demonstrable cholesterol-lowering effects observed in humans [30] would translate to the production of eggs with a lower cholesterol content. Specifically, we postulated that cholesterol-lowering effects were more likely if the diet contained isoflavones in the biologically active and bioavailable aglycon form rather than as isoflavone glycosides [28,50,51,52]. To test this, we compared diets that were essentially similar in composition (Table 1) but differed with regard to the qualitative and quantitative composition of isoflavones (Table 2).

White Leghorn hens are globally among the most popular commercial strains of egg-laying birds, characterized by early sexual maturity (3–4 mo) and a high rate of egg production, typically about 280 eggs/animal/year. However, after the first year the quantity and/or quality of eggs produced markedly declines in parallel with a decrease in endogenous estrogen production [36]. In this respect, the aging hen can be considered a potential animal model for a perimenopausal/postmenopausal status. Egg-laying typically declines by approximately 80% over this period and molting occurs, taking hens out of egg laying after 68–72 weeks of age despite being able to produce sufficient estrogen to sustain a low rate of egg production [36]. The hens used in our study were 72 weeks old and characterized by a very low egg-laying efficiency (37.8%) at the time of randomization into the diet groups.

Poultry worldwide are typically fed commercial diets composed of a mix of corn, soymeal and other nutrients that yield eggs with standard nutritional characteristics. Consequently, hens are exposed lifelong to a relatively high background level of dietary isoflavones, predominantly in the form of the glycoside conjugates of daidzein (daidzin) and genistein (genistin). This is also the case for most rodents fed commercial rodent chow that is typically formulated with soy protein [53]. These conjugated isoflavones are not bioavailable or biologically active, but rather, require conversion to bioactive aglycons by the action of intestinal β-glucosidases, which then facilitates absorption [28]. Further metabolism takes place in the intestine, and the hen [33,34], like most other animal species [5,54,55] except humans [2,3,12,13,14], consistently produces high concentrations of *S*-(-)equol, a bacterial metabolite with greater biological potency than its precursor, daidzein [29].

In this study, the source of the added isoflavone aglycons was a commercial soy germ pasta [30], shown in several clinical studies to have beneficial health effects [30,31,32]. It was finely ground and substituted 53.4% by weight for some of the corn and wheat bran in the standard commercial poultry diet (Table 1). Pure isoflavone standards were not used because of the prohibitive cost and difficulty of obtaining large quantities for this study design. The extruded soybean meal, which made up 21% of the standard poultry diet (Control), was retained in the test diets so that the contribution of isoflavone glycosides to the Control diet was similar to the other diets. Because isoflavones are highly concentrated in the soy germ [49,56], the inclusion of finely ground soy germ pasta contributed only 1.06% of soy germ to the diet (diet B), but it approximately doubled the total isoflavone content compared with the Control diet, and this increase was accounted for by the addition of predominantly aglycons (Table 2). A similar proportion of soy germ (with isoflavones in exclusively the glycoside form) was used in diet A to permit comparison between aglycons and glycosides. Finally, a group of hens was fed a diet made with conventional finely ground pasta lacking any soy germ or isoflavones (diet C) to rule out any effect from durum wheat in the pasta. The hens were compliant to the diets as evidenced from the monitoring of feed intake and body weight (Table 3).

Our data show that when isoflavone aglycon-enriched soy germ pasta was incorporated into a commercial poultry diet, it significantly reduced plasma cholesterol concentrations of laying hens, resulting in eggs with lower cholesterol content—the average reduction being approximately 30% when compared with eggs from the Control group. It is well established that soy protein modestly lowers serum cholesterol levels in humans [57], and this was the basis for the 1998 FDA-approved health claim that soy protein reduces cardiovascular risk [58]. However, at that time, the role of isoflavones was unclear due to a paucity of data. However, it was later shown that soy protein devoid of isoflavones had no cholesterol-lowering effect [59], and several meta analyses point to the importance of isoflavones in the reduction of serum cholesterol [60,61,62]. Given the broad range of biological activities of isoflavones and equol [29], there are potentially many possible mechanisms for the lipid-lowering effects. Isoflavones are good agonists for nuclear receptors [63,64], and FXR, which is the rate-limiting enzyme for bile acid synthesis in cholesterol, is a receptor critically involved in lipid and glucose metabolism. Molecules based on the isoflavone skeleton have been in development to treat dyslipidemia targeting this pathway [65]. We would speculate that this same mechanism may be a key factor that accounts for the reduced serum cholesterol concentrations in hens, which would inevitably lead to reduced egg-yolk cholesterol levels. This reduced cholesterol content was only partly related to a reduced yolk mass, due to the increase in the hen’s deposition rate (Figure 1 and Figure 2), because when expressed relative to yolk mass, eggs laid by these hens still had lower cholesterol contents. The reduced cholesterol content of the eggs was an important finding, especially given the relevance of eggs to the human diet and the long-standing association and controversies between cholesterol intake and cardiovascular risk [66,67].

Furthermore, in the present study, the changes in plasma and egg cholesterol levels were inversely proportional to both the plasma *S*-(-)equol concentration and to the egg *S*-(-)equol content.

The mean plasma concentration of *S*-(-)equol was almost 3-fold higher in hens fed the two diets containing soy germ where the isoflavone intake was doubled by increases in either glycosides (diet A) or aglycons (diet B). The highest plasma *S*-(-)equol concentrations were attained with the latter diet, a finding that supports the pharmacokinetics of isoflavones, as aglycons tend to yield higher plasma concentrations than glycosides [50,51,52], and validates our concept that *S*-(-)equol formation is more likely if the aglycon, daidzein, is ingested rather than its glycoside, daidzin, since the first hydrolysis step is not required [5]. Plasma *S*-(-)equol concentrations in all hens were in a similar range to that reported for humans consuming soy foods that are equol producers [14], although higher when adjusted for body weight. Our data show that isoflavones and *S*-(-)equol are readily transferred into the egg yolk, as has been previously reported in hens fed soy-based poultry diets [35,68,69], indicating that dairy products can be a source of low levels of isoflavones in the human diet [70].

An unexpected and remarkable finding from this study was the effect the diet containing isoflavone aglycons from soy germ pasta had on the egg-laying efficiency of the hens. The egg-laying efficiency of the hens among the dietary groups was similar (37.8%) at the start of the study, but over the subsequent 28-day study period, hens fed the Control corn/soymeal diet and those fed the same diet supplemented with isoflavone glycosides from soy germ (diet A), or conventional pasta with no isoflavones (diet C) all showed a similar natural weekly decline in the egg-laying rate, so that in the final week, the egg-laying rate decreased to 30.0, 27.1 and 30%, respectively, for these groups (Figure 1). By contrast, after the first week of acclimatization to the diet, the egg-laying efficiency of hens fed the diet supplemented with isoflavone aglycons (diet B) gradually increased, as shown in the “egg-array” depicting each day an egg was laid. In the final week, this group of hens had achieved an egg-laying rate of 64.3% (Figure 2). In total, over this 4-week period, 141 eggs were produced by hens fed this diet, which compared with only 87, 81 and 87 eggs from hens fed the Control diet, diet A and diet C, respectively. Therefore, despite a similar intake of isoflavones in hens fed diet A, which was mainly glycosides, no increase in egg production was observed. These data indicate that dietary isoflavone aglycons, and not glycosides, improve the egg production of hens by 62–74% in hens that otherwise would be expected to show gradual declining rates of egg laying. It is possible that the higher plasma *S*-(-)equol concentrations may be a cause of the greater egg-laying efficiency of the hens fed ground soy germ pasta. It has been reported that synthetic daidzein increased egg production in Shaoxing ducks and white silky fowls during their late period of the laying cycle [71,72], but interestingly, this effect was not observed during the early stage of duck reproduction [73]. We interpret these observations and the results from our study to imply that dietary isoflavone aglycons may have a greater agonist effect in low estrogenic states that occur in the late period of laying. The impressive increase in the production of eggs by hens fed the diet supplemented with isoflavone aglycons from soy germ pasta occurred alongside a concomitant increase in food intake (33.6 vs. 27.9, 26.4 and 28.2 Kg, respectively, for the Control group and diet groups A and C), probably due to the active laying period of such hens. Interestingly, we observed no effect on the egg-laying rate in hens with a plasma *S*-(-)equol concentration of <300 ng/mL, whereas above this threshold, the egg-laying rate increased. This suggests that there may be a threshold plasma *S*-(-)equol concentration for an estrogenic effect mediated through ERβ, because *S*-(-)equol has selective affinity for this estrogen receptor [4,74,75]. The underlying mechanism by which isoflavones or *S*-(-)equol influence ovulation, and hence, egg laying, remains unclear. It was recently suggested that daidzein influences the avian laying performance via the modulation of mRNA expression of gonadotropin receptors in ovarian follicles, especially during the late periods of the reproductive cycle [76]. Gonadotropins are the primary regulators of follicular growth and ovulation, and their biosynthesis and actions are both regulated by estrogens [71]. Although it was outside the scope of this study to examine the mechanism for the increased egg-laying rate in hens given the diet supplemented with isoflavone aglycons, it is possible that *S*-(-)equol may have a more potent effect than daidzein in conditions of a low estrogen status.

The formation of an egg requires a major reproductive effort by the hen and involves the conversion of nutrients in the feed into egg constituents; consequently, the diet influences the final chemical composition of the egg [36]. Significant differences were observed in the egg-yolk color of the eggs laid by hens fed both pasta-containing diets. These egg yolks were less yellow as measured on the Roche color scale (Table 4). This difference merely reflects the substitution of some of the corn carotenoids with the finely ground pasta. The quality of the eggs laid, as measured on the Haugh scale, was similar among the four groups. However, the composition of the eggs was slightly altered by the addition of isoflavone aglycons from the ground soy germ pasta. Most notably, the eggs from this group of hens had an increased albumen mass and decreased yolk mass, leading to a higher albumen/yolk ratio when compared with eggs produced from hens fed the Control diet, or the other diets that delivered isoflavone glycosides. This finding concurs with a decreased yolk/albumen ratio previously reported in eggs from ducks (*Anas platyrhynchos*) treated with 5 mg/Kg of synthetic daidzein for 9 weeks [71]. Estrogen is known to regulate albumen synthesis in the oviduct and yolk deposition is inversely proportional to the egg-laying rate [36]. Whether it is the higher plasma equol concentrations in these hens, the increased egg-laying rate, or both that explain the change in the albumen/yolk ratio is unclear. It may be that this is an estrogenic effect with equol increasing the number of mature oocytes, leading to the production of numerous eggs with a reduced yolk mass.

## 5. Conclusions

In conclusion, the previously described clinical effects of a novel soy germ pasta containing predominantly isoflavone aglycons [30] have now been recapitulated in the aging Leghorn hen. The effect of enriching the diet with isoflavone aglycons on the hen’s plasma cholesterol was the production of eggs with a lower cholesterol content that was inversely correlated with plasma *S*-(-)equol concentration and egg *S*-(-)equol content. Impressively, an increase in the egg-laying rate of aging hens at a time when there is typically a natural decline in egg production was a key finding that appeared to be mediated through the conversion of daidzein to *S*-(-)equol. None of these effects were observed with the dietary substitution of comparable amounts of isoflavone glycosides from soy germ, or with the substitution of durum wheat in a conventional pasta devoid of isoflavones. These findings attest to the greater biological activity and efficacy of a diet containing predominantly isoflavone aglycons. The implication of these findings for the industry is the potential to stabilize and extend the reproductive lifetime of hens, and to produce eggs with the unique characteristics of lower cholesterol content with potential beneficial effects for human health.

## Figures and Tables

**Table 1:** Ingredient and nutrient composition of the Control diet, the Control diet with added soy germ providing mainly isoflavone glycosides (diet A), the Control diet with addition of ground dried soy germ pasta with predominantly isoflavone aglycons (diet B), and the Control diet with addition of conventional pasta lacking isoflavones (diet C).

Ingredients (% of Diet):	Control Diet	Diet A	Diet B	Diet C
Corn meal	51.6	51.6	10.0	10.0
Extruded soybean meal, 44%	21.0	20.1	19.0	19.0
Corn gluten meal	8.0	8.0	6.5	7.6
Wheat bran	10.5	10.5	-	
Durum wheat semolina	-	-	53.4 ^†^	53.4
Sunflower oil	0.4	0.4	1.5	1.5
Dicalcium phosphate	0.5	0.5	0.5	0.5
Calcium carbonate	5.5	5.5	5.5	5.5
Sodium bicarbonate	0.5	0.5	0.5	0.5
NaCl	0.5	0.5	0.5	0.5
Vitamin and mineral premix *	1.5	1.5	1.5	1.5
Soy germ	-	0.9	1.06	-
**Nutrient composition** **(% dry matter):**				
Dry matter	88.2	88.2	84.1	89.4
Crude protein	18.6	18.8	18.7	18.5
Ether extract	3.7	3.9	3.6	3.5
Crude fiber	5.6	5.6	5.4	4.7
Ash	13.6	13.6	12.1	11.5
Estimated metabolizable energy (MJ/kg)	11.5	11.6	11.4	11.4

* Provided per kilogram of diet: vitamin A, 12,500 IU; cholecalciferol, 3000 IU; DL-alpha-tocopheryl acetate, 60 mg; vitamin B_1_, 2 mg; vitamin B_2_, 6 mg; vitamin B_6_, 4 mg; pantothenic acid, 8 mg; PP, 30 mg; folic acid, 0.50 mg; vitamin B_12_, 0.02 mg; vitamin K, 2 mg; choline, 750 mg; Fe, 35 mg; Zn, 42 mg; I, 0.5 mg; Co, 0.5 mg; ^†^ ingredients of soy germ pasta before the blending are published elsewhere [30].

**Table 2:** Comparison of the isoflavone composition of the diets fed to aging White Leghorn hens (values represent the mean ± SD of duplicate measurements).

Isoflavone, mg/100 g	Control Diet	Diet A	Diet B	Diet C
Daidzein forms	12.2 ^a^ ± 2.12	20.5 ^b^ ± 3.01	24.5 ^b^ ± 2.17	10.5 ^a^ ± 1.85
Genistein forms	17.9 ^a^ ± 1.14	30.1 ^b^ ± 3.04	32.6 ^b^ ± 2.79	16.1 ^a^ ± 2.17
Glycitein forms	3.4 ^a^ ± 0.64	5.7 ^a^ ± 1.02	6.3 ^b^ ± 0.02	3.1 ^a^ ± 1.81
Total isoflavone (aglycon equivalents)	33.6 ^a^ ± 3.12	56.4 ^b^ ± 4.07	63.0 ^b^ ± 2.84	30.4 ^a^ ± 2.40
Proportion of glycosides (%)	95.0 ^b^	96.8 ^b^	49.8 ^a^	96.7 ^b^
Proportion of aglycons (%)	5.0 ^a^	4.2 ^b^	50.2 ^a^	4.3 ^b^

^a^, ^b^, *p* < 0.05: values with different letters in the same row represent significant differences.

**Table 3:** Summary of egg productivity over a 4-week period of aging White Leghorn hens (*n* = 10/group) fed a commercial corn/soymeal poultry diet (Control), the same diet supplemented with soy germ to increase the content of isoflavone glycosides (diet A), the same diet with added milled soy germ pasta containing predominantly isoflavone aglycons (diet B) and the same diet with conventional pasta lacking isoflavones (diet C).

Hens’ Egg Productivity	Control Diet	Diet A	Diet B	Diet C
Total number of eggs laid (*n*)	87	81	141	87
Average egg weight (g)	52.2 ± 1.7	55.6 ± 1.7	52.9 ± 2.1	54.0 ± 1.8
Total egg yield ^x^ (g)	4541	4504	7459	4699
Baseline egg-laying rate (%)	37.8 ± 6.8	37.8 ± 6.8	37.8 ± 6.8	37.8 ± 6.8
Final egg-laying rate (%)	30.0 ± 4.5	27.1 ± 8.1	64.3 ^a^ ± 15.4	30.0 ± 15.4
Total egg-laying rate ^y^ (%)	31.1 ± 5.6	28.9 ± 8.8	50.4 ± 15.3	31.1 ± 10.2
Total food consumption (kg)	29.8	28.9	33.6	30.7
Baseline body weight (kg)	1.81 ± 0.19	1.88 ± 0.28	1.82 ± 0.25	1.78 ± 0.17
Final body weight (kg)	1.75 ± 0.24	1.79 ± 0.20	1.93 ± 0.27	1.72 ± 0.22

Values are expressed as mean ± SEM. ^a^ *p* < 0.05 when compared with all other groups in the same row. ^x^ (total number of laid eggs) × (average egg weight in g); ^y^ (total number of laid eggs) × 100/(total experiment days) × (total number of hens).

**Table 4:** The compositional analysis and quality assessment of eggs (*n* = 10 per group) laid by hens fed a commercial corn/soymeal poultry diet (Control), the same diet with added isoflavone glycosides from soy germ (diet A), the same diet with isoflavone aglycons from milled soy germ pasta (diet B) and the Control diet with commercial pasta devoid of isoflavones (diet C).

	Control Diet	Diet A	Diet B	Diet C
**Egg Constituents and Quality Markers:**				
Albumen (% of weight)	53.0 ^a^ ± 0.4	55.2 ^a^ ± 0.6	57.1 ^b^ ± 1.1	55.9 ^a^ ± 0.5
Yolk (% of weight)	35.9 ^b^ ± 1.0	33.8 ^b^ ± 0.8	31.9 ^a^ ± 0.6	33.5 ^b^ ± 0.3
Albumen/yolk ratio	1.6 ^a^ ± 0.1	1.6 ^a^ ± 0.1	1.7 ^b^ ± 0.1	1.6 ^a^ ± 0.1
Shell (% of weight)	11.1 ± 1.1	10.9 ± 0.7	11.2 ± 0.5	11.0 ± 0.5
Haugh unit	78.1 ± 4.7	79.4 ± 7.5	75.9 ± 6.7	75.4 ± 4.6
Roche color scale	12.0 ^a^ ± 1.0	13.0 ^a^ ± 1.0	6.0 ^b^ ± 1.0	6.0 ^b^ ± 1.0
**Yolk Constituents:**				
Dry matter (% of wet yolk)	50.4	50.6	51.0	51.0
Ether extract (% of wet yolk)	32.3	32.1	31.8	32.1
Crude protein (% of wet yolk)	16.0	15.8	15.8	15.8
Ash (% of wet yolk)	1.6	1.7	1.7	1.7
Cholesterol, mg/g egg yolk	14.6 ^b^ ± 0.7	14.0 ^ab^ ± 0.7	13.5 ^a^ ± 0.6	14.8 ^b^± 0.4
Cholesterol content, mg/egg	275 ^b^ ± 18	262 ^b^ ± 19	220 ^a^ ± 15	266 ^b^ ± 9

^a^, ^b^, *p* < 0.05: values with different letters in the same row indicate significant differences.

## Data Availability

Data generated in this study and supporting the reported results are retained by C.C. at the Department of Agricultural, Environmental and Food Science, University of Perugia, Borgo XX Giugno 74, 06124, Perugia, Italy. and are available upon written request.
